# Environmental regulations, green innovation and intelligent upgrading of manufacturing enterprises: evidence from China

**DOI:** 10.1038/s41598-020-71423-x

**Published:** 2020-09-02

**Authors:** Fansheng Meng, Ye Xu, Gang Zhao

**Affiliations:** School of Economics and Management, Harbin Engineering University, Harbin, 150001 China

**Keywords:** Environmental sciences, Environmental social sciences

## Abstract

Intelligent development is an inevitable choice for the green development of manufacturing enterprises, environmental regulations and green innovation play an important role in the intelligent development of manufacturing enterprises. By analyzing the interrelationships between environmental regulations, green innovation, environmental dynamism and the intelligent upgrading of manufacturing enterprises, this paper builds a model of the impact of environmental regulations on the intelligent upgrading of manufacturing enterprises. Taking the listed Chinese manufacturing enterprises that implement intelligent upgrades as the survey object, the concept model was verified. The results of the study show that environmental regulations, green innovation have a significant positive effect on intelligent upgrading of manufacturing enterprises. Furthermore, it reveals that green innovation plays a partially mediating role in the relationship between environmental regulations and the intelligent upgrading of manufacturing enterprises. Environmental dynamism have a positive adjustment effect between green innovation and the intelligent upgrading of manufacturing enterprises. The conclusion of the study deepens the relevant research on the intelligent upgrade of manufacturing enterprises, and provides theoretical basis and reference for the intelligent development of manufacturing enterprises.

## Introduction

Environmental protection and green development are hot issues of common concern to all countries in the world. Manufacturing enterprises will produce negative externalities when they produce. The environmental pollution caused by production needs to be solved urgently^1^. With the German “Industry 4.0”, the US “Re-industrialization” and China’s “Made in China 2025” strategies successively proposed, countries around the world have taken intelligent upgrading as the main direction and goal of green sustainable development of manufacturing enterprises. In order to cope with the pressure and challenges of the external environment, it is an important way for manufacturing enterprises to carry out intelligent upgrades^2,3^. At the same time, in order to cope with the constraints and requirements of internal and external factors such as environmental regulations, manufacturing enterprises need to carry out green innovation. However, is there any mutual influence or a certain relationship between environmental regulations, green innovation and intelligent upgrading. Whether there is a contradiction between environmental regulations and intelligent upgrading. It has become an important issue faced by manufacturing enterprises in promoting intelligent upgrading. The research on the impact of environmental regulation on the intelligent upgrading of manufacturing enterprises mainly focuses on the relationship between environmental regulations and green innovation. However, there are relatively few studies on the relationship between environmental regulations and the intelligent upgrading, and between green innovation and intelligent upgrading. Most of the existing research is qualitative analysis and industry analysis. In addition, many scholars have inconsistent views. Some scholars believe that environmental regulations can promote the intelligent upgrading of enterprises. Other scholars pointed out that environmental regulations will cause enterprises to invest more costs to maintain the survival of enterprises. As a result, enterprises do not have more capital to invest in intelligent upgrades. Not conducive to the intelligent upgrade of enterprises^4^.

Based on the perspective of manufacturing companies, through theoretical analysis of environmental regulations, green innovation and intelligent upgrade, this article proposes relevant research assumptions. The relationship model of environmental regulations, green innovation, environmental dynamism and intelligent upgrade is established. Based on the data of Chinese manufacturing listed companies in the process of intelligent upgrade, this article quantitatively analyzes the relationship between environmental regulations, green innovation, environmental dynamism and intelligent upgrading. This article quantitatively studies the role of environmental regulations in promoting intelligent upgrading. It proves the mediating role of green innovation between environmental regulations and intelligent transformation and upgrading. The mediating role of environmental dynamics between green innovation and intelligent upgrading is discussed. The article strives to provide theoretical guidance and practical inspiration for the intelligent upgrading of manufacturing enterprises.

The possible marginal contributions of this paper are as follows: Firstly, the impact mechanism of environmental regulations on the intelligent upgrade of manufacturing enterprises is clarified. By constructing a mechanism model of environmental regulations on the intelligent upgrading of manufacturing enterprises, the direct and indirect effects of environmental regulations on the intelligent upgrading of manufacturing enterprises are analyzed. Secondly, it reveals the important role of green innovation in environmental regulations and intelligent upgrading of manufacturing enterprises. By analyzing the relationship between green innovation and the intelligent upgrading of manufacturing enterprises, green innovation and environmental regulations, it verifies the intermediary role of green innovation in environmental regulations and intelligent upgrading of manufacturing enterprises. Thirdly, clarify the regulatory role of environmental dynamism between green innovation and the intelligent upgrading of manufacturing enterprises. This article analyzes the relationship between environmental dynamism and green innovation and the intelligent upgrade of manufacturing companies, and empirically examines the regulatory role of environmental dynamism between green innovation and the intelligent upgrade of manufacturing companies.

The rest of this article is organized as follows. “Literature review” section summarizes and reviews relevant important literature. “Research hypotheses” presents research hypotheses among environmental regulations, green innovation, environmental dynamism, and intelligent upgrades. “Materials and method” section presents the research design and method of this paper. “Empirical results and discussion” section verifies the research hypothesis, gives the research results, and discusses the results. “Conclusion and implications” section draws conclusions and theoretical implications.

## Literature review

### Environmental regulations

Environmental regulations is a series of related policies or measures adopted by the government to protect the environment^5^, Environmental regulations have effectively restricted the destruction of enterprises by the environment and played an important role in protecting the environment^6,7^. From the perspective of the enterprise, scholars have mainly studied the impact of environmental regulations on enterprises. They believe that appropriate environmental regulations can promote enterprises to establish relative competitive advantages, and are also an important driving force for enterprises to improve performance^8,9^. However, environmental regulations have increased the operating costs of enterprises, which may affect the operating conditions of enterprises and are not conducive to the growth of performance^10,11^.

### Green innovation

At present, with regard to the research on the connotation of green innovation, most scholars mainly start from the content and objectives of green innovation. From the perspective of content, Chen and other scholars^12^ believe that green innovation is an innovation related to green production processes or green products, including pollution prevention, energy saving, waste utilization, environmental management and product design. From the perspective of goals, scholars such as Driessen^13^ believe that green innovation is an innovation that can greatly improve environmental benefits. Based on the above analysis, this article believes that green innovation is an innovation that enterprises carry out in the prevention and control of pollution, product design, and waste utilization in the production process or enterprise products in order to reduce the adverse impact of production and operation activities on the external environment. Based on the enterprise perspective, the current research on green innovation generally includes two aspects. On the one hand, it is about the dynamic factors of enterprise green innovation. Stakeholder theory believes that stakeholders such as suppliers, consumers and competitors of enterprises are the driving force for enterprises to carry out green innovation^14^, Institutional theory holds that the pressure of environmental regulations is the driving force for enterprises to carry out green innovation^15^, Natural resource theory believes that organizational resources, enterprise capabilities, and the importance of managers are important factors that drive enterprises to carry out green innovation^16^. On the other hand is the impact of green innovation on business performance. Some scholars have suggested that green innovation will increase the enterprise's resource investment, make the already limited enterprise resources more scarce, and will not be conducive to the increase of enterprise performance^17^, Scholars such as Porter believe that green innovation by enterprises can not only improve the utilization rate of resources of enterprises, but also reduce the production costs of enterprises, thereby increasing the performance and income of enterprises^18^.

### Intelligent upgrade

Scholars at home and abroad mainly study the intelligent upgrading of manufacturing enterprises from the aspects of influencing factors, models and paths. From the perspective of influencing factors, the factors that affect the intelligent upgrading of enterprises can be divided into technical factors, enterprise factors, market structure factors, etc.^19^. For manufacturing enterprises, digital integration and technological innovation are the main technical reasons affecting their intelligent upgrade^20^, Enterprise factors mainly refer to the ability to independently arrange production according to the actual needs of users, flexible production planning and scheduling, etc.^21^, In particular, the profitability of the enterprise, the current level of intelligence in the industry, and labor costs will significantly affect the intelligent upgrade of the enterprise^22^, The product market demand, interaction ability, etc. are the market structure factors that affect the intelligent upgrade of the enterprise^11^. From the perspective of the intelligent upgrade mode, based on the information physical interaction perspective, the intelligent upgrade mode can be divided into three levels, namely, intelligent platform, horizontal integration and vertical integration^23^; Based on the intelligent development process of manufacturing industry, the intelligent upgrade model can be divided into three basic paradigms, namely digital manufacturing, digital networked manufacturing and a new generation of intelligent manufacturing^24^; From the perspective of emerging models, the intelligent upgrade model can be divided into cloud manufacturing, Ubiquitous Information Manufacturing, smart manufacturing, active manufacturing, manufacturing IoT, social enterprises, etc.^25^. In addition, the more widely used model of manufacturing enterprises is the intelligent manufacturing sharing model^26^. From the perspective of the intelligent upgrade path, many documents believe that the intelligent development of manufacturing enterprises should adopt the technology upgrade path of parallel promotion and integration^24,27,28^. And take core technology, production methods, standard systems, etc. as an important path for intelligent upgrading of manufacturing enterprises^3,29^. At the same time, in order to achieve an investment-driven shift to an element-driven, from resource consumption to green development, from simple manufacturing to smart manufacturing, a new generation of information technology and advanced manufacturing technology should be combined to cultivate an intelligent upgrade path for manufacturing enterprises^29^.

In recent years, the impact of intelligent upgrading on employment has become a hot issue. One view is that the intelligent upgrading of manufacturing companies will replace employment. Acemoglu and Restrepo^30^ found that manufacturing intelligence has replaced labor employment to a certain extent, adding one robot per thousand people will reduce the number of employed population by 0.18% to 0.34%, and reduce per capita wages by 0.25% to 0.5% . The research of Dekker et al.^31^ also got a similar view. Another view is that the intelligent upgrading of manufacturing enterprises will not replace employment. Borland and Coelli^32^ found that the development of computer technology did not reduce the workload, the structure of the labor market and the speed of work turnover did not accelerate, and questioned the thesis of the "employment destruction effect of technological progress". Morikawa^33^ analyzed the original survey data of more than 3,000 Japanese companies and found that employees with higher education tend to expect artificial intelligence and other related technologies to have a positive impact on their employment. In addition, there is a view that there are multiple consequences for the impact of the intelligent upgrading of manufacturing enterprises on employment. Acemoglu and Restrepo^34^ constructed a theoretical model of the impact of artificial intelligence and automation on employment. Research has shown that the employment substitution effect of automation will reduce labor demand and wages, but it will be offset by the productivity effect. Automation brings cost savings and capital accumulation. Will increase labor demand, and automation will have different effects on high-skilled labor and low-skilled labor.

### Research on the relationship between environmental regulations, green innovation and intelligent upgrade

Some scholars have studied the relationship between environmental regulations and green innovation. Ren et al.^35^ believe that environmental regulations is an important factor influencing green innovation, Using the panel data of Chinese manufacturing enterprises from 2011 to 2015, it is concluded that environmental regulations will positively affect the green innovation of manufacturing enterprises. Aldieri et al.^36^ proposed that green innovation is an important method for companies to deal with environmental pressures, and there is an important relationship between knowledge procurement strategies and green innovation. Xie et al.^37^ divided environmental regulations into command-controlled environmental regulations and market-incentive environmental regulations. They believed that command-controlled environmental regulations had a double-threshold effect on green growth, and market-driven environmental regulations had a single-threshold effect on green growth. At the same time, Peuckert^38^ believes that environmental regulations has time heterogeneity for green technology innovation. In the short term, environmental regulations has a negative impact on green technology innovation, and in the long term, environmental regulations has a positive impact on green technology innovation. Song et al.^39,40^ found that environmental regulations can not only reduce environmental pollution, but also effectively promote the enhancement of enterprises' technological innovation capabilities. Zhang et al.^41^ found that the upgrading of industrial structure can reduce haze pollution, at the same time, environmental regulations can play a role in promoting.

Some scholars paid attention to the relationship between technological innovation and intelligent upgrading. Some scholars have researched and proposed that technological innovation can promote the intelligent upgrade of manufacturing enterprises, and technological innovation is an important driving force for the intelligent upgrade of manufacturing enterprises^24^. However, green innovation will cause enterprises to generate additional investment in innovation^42^, that is to say, relative to technological innovation, green innovation will increase the production cost of enterprises, whether green innovation will affect the intelligent upgrade of manufacturing enterprises, and how much impact, is still worthy of further research.

Environmental regulations has an important impact on the development of manufacturing enterprises, and intelligent upgrade is an important direction for the green development of manufacturing enterprises. environmental regulations is very important for the intelligent upgrading of manufacturing enterprises, and is related to the green sustainable development of manufacturing enterprises. However, at present, there is relatively little research on the relationship between environmental regulations and intelligent upgrade. Only the qualitative research on the important impact of intelligent manufacturing enterprises on environmental sustainable development^43^, The relationship between environmental regulations and the intelligent upgrade of manufacturing enterprises, green innovation and the intelligent upgrade of manufacturing enterprises has not yet been clarified. Therefore, it is necessary to incorporate environmental regulations, green innovation and intelligent upgrading of manufacturing enterprises into a research framework, and systematically analyze the relationship between environmental regulations, green innovation and intelligent upgrade of manufacturing enterprises.

## Research hypotheses

### Environmental regulations and intelligent upgrade of manufacturing enterprises

On the one hand, under the constraints of environmental regulations, manufacturing enterprises are willing to undergo transformation and upgrading. Mainly due to the following reasons, one is that environmental regulations have increased the production costs of manufacturing enterprises. In order to reduce costs, manufacturing enterprises hope to make appropriate changes to compensate for the increased costs of enterprises due to environmental regulations^44^. The second is the higher standards of environmental protection requirements imposed on manufacturing enterprises by environmental regulations, which has intensified competition among manufacturing enterprises in the industry and formed the survival of the fittest. In order to maintain the survival and development of enterprises, manufacturing enterprises will be more willing to carry out transformation and upgrading to enhance their own comprehensive strength^45^. Third, environmental regulations have enhanced the willingness of enterprises to seek more benefits, and enterprises are willing to carry out transformation and upgrading in order to seek more profits. At the same time, existing studies have shown that environmental regulations can effectively promote industrial upgrading under certain circumstances^46^. It can be seen that manufacturing enterprises are more willing to carry out transformation and upgrading under the constraints of environmental regulations.

On the other hand, manufacturing enterprises are more inclined to take intelligence as the direction of transformation and upgrading. First of all, the release of intelligent related policies will enable enterprises to enjoy many preferential policies and supportive measures for intelligent upgrading. Secondly, the development of new-generation information technology such as artificial intelligence is very rapid, and has the conditions for commercialization, The integration of next-generation information technology such as artificial intelligence with advanced manufacturing technology to achieve intelligent upgrading of manufacturing enterprises is an important path for manufacturing enterprises to transform and upgrade^47^. Finally, the needs of the market and customers are diversified and personalized. The intelligent upgrade of manufacturing enterprises can meet the personalized and diversified needs of the market and customers, and can increase the profits of enterprises, which is in line with the development direction of manufacturing enterprises^48^. It can be seen that manufacturing enterprises choose to upgrade intelligently will save costs and increase corporate profits. Therefore, manufacturing enterprises will choose to upgrade intelligently.

In summary, environmental regulations can increase the willingness of manufacturing enterprises to upgrade intelligently, and make manufacturing enterprises willing to invest more costs for intelligent development. Therefore, this article proposes the following hypothesis:

#### Hypothesis H1

Under the condition that other conditions remain unchanged, environmental regulations can positively affect the intelligent upgrading of manufacturing enterprises.

### Environmental regulations and green innovation

Environmental regulations is an important factor for manufacturing enterprises to implement green innovation, mainly in the following aspects, First of all, Porter’s theory that environmental regulations can encourage enterprises to carry out more R & D and innovation activities, and enhance their technological capabilities and overall competitiveness^18^. Scholars such as Yang believe that environmental regulations can promote green innovation by enterprises through the compensation effect of innovation^49^. Secondly, environmental innovation can regulate the relationship between environmental regulations and environmental performance, and provide valuable guidance for corporate managers to formulate environmental innovation strategies^50^. In order to reduce pollution and comply with the relevant requirements of environmental regulations, manufacturing enterprises will improve their pollutant discharge capacity and reduce environmental pollution through green technology R & D and other means. This move not only enhances the enterprise’s environmental protection capabilities, but also promotes green innovation in the production process and products. Thirdly, although environmental regulations will have an adverse effect on enterprises in a short period of time, they may promote technological innovation^51^. environmental regulations have increased the cost of enterprises, And through green innovation, enterprises can increase profits without polluting the environment, In this way, under the premise of complying with environmental regulations, enterprises not only increase profits but also promote their green innovation capabilities.

In summary, under the environmental regulations, manufacturing enterprises will accelerate R & D investment and promote green innovation in order to reduce pollution and increase profits. Therefore, this article proposes the following hypothesis:

#### Hypothesis H2

With other conditions remaining unchanged, environmental regulations can positively affect green innovation in manufacturing enterprises.

### The mediating effect of green innovation

Green innovation and intelligent upgrading of manufacturing enterprises. The intelligent upgrade of manufacturing enterprises mainly includes intelligent production methods, equipment intelligence and product intelligence^29^. This article believes that the relationship between green innovation and the intelligent upgrading of manufacturing enterprises mainly includes three aspects. First, green innovation and intelligent production methods. In terms of production methods of manufacturing enterprises, green innovation will bring about changes in production methods, personnel organization and production structure of manufacturing enterprises. This allows manufacturing enterprises to adapt to the changing needs of users in a timely manner, and to streamline redundant and unnecessary processes in the production process^29^, and to improve production efficiency. At the same time, it meets the ever-changing needs of users, thus accelerating the process of enterprise intelligence in the production process. Second, green innovation and equipment intelligence. The intelligentization of manufacturing equipment is the basis and premise for enterprises to realize intelligent manufacturing. Green innovation drives the innovation of production technology. Promote the transformation of manufacturing equipment from traditional equipment to digital, networked and intelligent equipment, thus promoting the intelligent development of manufacturing equipment to a certain extent. Finally, green innovation and product intelligence. The products produced by traditional manufacturing enterprises are homogeneous, and green innovation can promote product innovation on the basis of improving product quality and output. And promote the product toward diversification and personalization, so that the product has more technological content, which is conducive to the intelligent upgrade of manufactured products^20^. Therefore, this article proposes the following hypothesis:

#### Hypothesis H3

Under the condition that other conditions remain unchanged, green innovation can positively affect the intelligent upgrade of manufacturing enterprises.

The intermediary role of green innovation in environmental regulations and intelligent upgrading of manufacturing enterprises, Based on the above analysis, we can see that environmental regulations can promote the intelligent upgrade of manufacturing enterprises, and environmental regulations can effectively promote the green innovation of manufacturing enterprises. At the same time, green innovation can also promote the intelligent upgrade of manufacturing enterprises. This also shows that environmental regulations is an important driving force for intelligent upgrading, which can not only directly affect the intelligent upgrading of manufacturing enterprises, but also indirectly promote the intelligent upgrading of manufacturing enterprises by promoting green innovation of enterprises. Green innovation is an important way for environmental regulations to affect the intelligent upgrade of manufacturing enterprises. In summary, under the influence of environmental regulations, green innovation has played a positive role in promoting the intelligent upgrading of manufacturing enterprises. Therefore, this article proposes the following hypothesis:

#### Hypothesis H4

While other conditions remain unchanged, green innovation has an intermediary role between environmental regulations and intelligent upgrading of manufacturing enterprises.

### The moderating effect of environmental dynamism

Environmental dynamism refers to the frequency and extent of changes in the internal and external environment and market demand faced by enterprises, as well as the unpredictability of changes^52^, Including various factors such as products and services and the degree of change of stakeholders such as customers and cooperative enterprises. High dynamic environment means that the market demand faced by the enterprise and the degree and frequency of changes in the internal and external environment are relatively large, otherwise, it is called a low dynamic environment^53^. The dynamic nature of the environment has increased the importance of exploration and innovation in manufacturing enterprises^54^. In a highly dynamic environment, with green innovation remaining unchanged, there are relatively many opportunities for manufacturing enterprises to upgrade to intelligence. These opportunities increase the probability of success for manufacturing enterprises to upgrade to intelligence. At the same time, when faced with a highly dynamic environment, according to prospect theory, when faced with potential losses, decision makers are more inclined to take risky decisions in order to reduce losses or increase income^55^, It can be seen that in a highly dynamic environment, the decision-makers of manufacturing enterprises are more willing to actively participate in the intelligent upgrade of enterprises. In a low dynamic environment, based on the fact that green innovation remains unchanged, manufacturing enterprises face relatively few opportunities for intelligent upgrades. Intelligent upgrades for manufacturing enterprises are a process of steady accumulation and gradual improvement. At the same time, when faced with a low dynamic environment, according to the prospect theory, when faced with stable and certain returns, corporate decision makers are more inclined to take conservative decisions in order to avoid risks^55^. It can be seen that under low dynamic environment, the decision-makers of manufacturing enterprises are not willing to upgrade their enterprises intelligently. Therefore, this article proposes the following hypothesis:

#### Hypothesis H5

Environmental dynamism have a positive adjustment effect between green innovation and the intelligent upgrading of manufacturing enterprises, With green innovation remaining unchanged, the higher the environmental dynamism, the greater the positive impact of green innovation on the intelligent upgrading of manufacturing enterprises.

**Figure 1:**
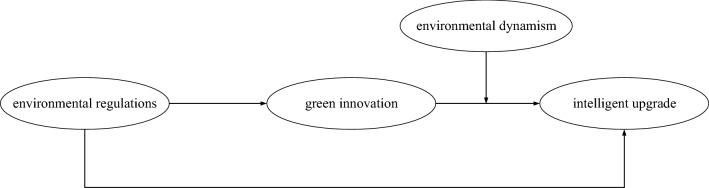
Conceptual model.

To sum up, based on the analysis and assumptions on the relationship between environmental regulations, green innovation, environmental dynamism and intelligent upgrading of manufacturing enterprises, this paper builds models of environmental regulations, green innovation, environmental dynamism and intelligent upgrading of manufacturing enterprises, as shown in Fig. 1.

## Materials and method

### Samples and data collection

In order to promote the implementation of the "Made in China 2025" strategy, From 2015 to 2018, the Ministry of Industry and Information Technology of China selected 305 enterprises nationwide as pilot enterprises for smart manufacturing. Because the data of listed enterprises is relatively complete and easy to obtain, based on the availability of data, this paper selects the enterprises listed in the Shanghai and Shenzhen stock markets as the research sample among the intelligent manufacturing pilot enterprises. The smart manufacturing pilot has been implemented since 2015, and the latest data is up to 2018. Among the smart manufacturing pilot enterprises, there are 59 enterprises listed on the Shanghai and Shenzhen stock exchanges. Excluding stocks with severe data loss and special handling (ST) and risk of delisting (* ST), In this paper, 52 listed enterprises are selected, and their data for 4 consecutive years are selected, with a total of 204 effective observations.

Data Sources. The data of "green invention patents" and "green utility model patents" used to measure green innovation come from Chinese Patent Full-text Database (CPFD) and are obtained through query and manual sorting. The environmental protection input data comes from the annual reports and social responsibility reports of listed enterprises. The names disclosed mainly include environmental protection expenditures, environmental protection inputs, greening environmental protection fees, and pollution control support. Control variables such as the size of the enterprise, the age of the enterprise, and the asset-liability ratio are all from China Stock Market & Accounting Research Database (CSMAR). At the same time, in order to prevent the abnormal value of each variable from affecting the estimation efficiency, the two ends of the variables with large differences were subjected to 0.95 and 0.05 tailing treatment.

### Research methods and variable measures

#### Research methods

**Figure 2:**
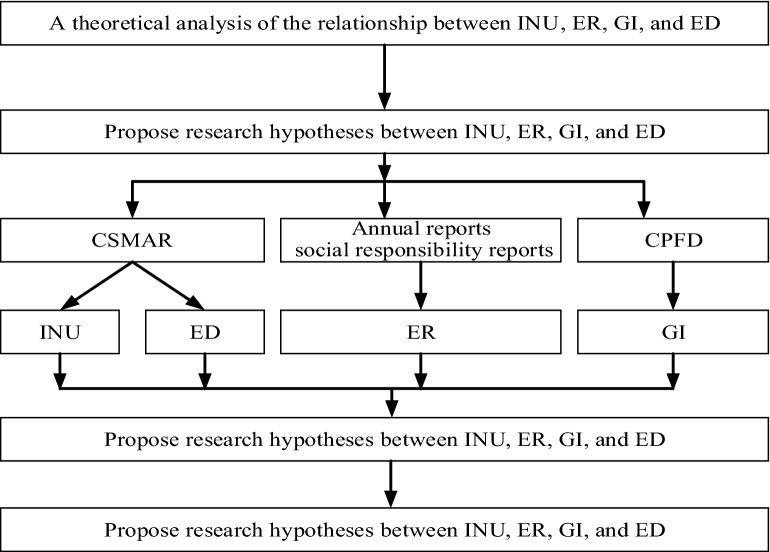
Flow chart.

Based on the analysis of the relationships among Intelligent Upgrade (INU), Environmental regulations (ER), Green Innovation (GI), and Environmental dynamism (ED), this paper proposes research hypotheses among INU, ER, GI, and ED. The data in China Stock Market & Accounting Research Database (CSMAR), Chinese Patent Full-text Database (CPFD), annual reports and social responsibility reports of listed companies are used. Constructed the correlation model between variables, and analyzed the correlation between variables. The research method is shown in Fig. 2.

#### Dependent variable "intelligent upgrade (INU)"

Since the enterprises selected in this article are all pilot enterprises for smart manufacturing identified by the Ministry of Industry and Information Technology of China, all the enterprises in the sample are enterprises in the process of intelligent transformation. Effectively ensure that the selected enterprises are in the process of intelligent upgrade. As for the measurement indicators of enterprise transformation and upgrading, scholars mostly use indirect indicators for alternative measurements. With regard to alternative variables for transformation and upgrading, Szirmai et al.^56^ and Ren et al.^57^ proposed that the result of enterprise transformation and upgrading, one of which is product transformation, is generally measured by the company's new product sales, and the proportion of new product sales in total product sales (NPS) can be used as a substitution variable. The other is the transformation of production methods or production efficiency of enterprises. The transformation of production methods or efficiency will lead to the transformation of products from low added value to high added value. The essence is that there are differences in the productivity of enterprises, and the total factor productivity (TFP) of enterprises can be used as a substitute variable. The intelligent upgrading of manufacturing enterprises studied in this article refers to the transformation of production methods or products brought to listed enterprises through the use of advanced manufacturing technology, artificial intelligence and other new-generation information technologies. It includes both the transformation of production methods and the transformation of enterprise products, Therefore, this paper uses two alternative indicators to measure the intelligent transformation and upgrading of manufacturing enterprises, namely the proportion of NPS and TFP.

Enterprise TFP calculation. There are various methods for calculating the enterprise's total factor productivity. This article is based on the research results of Jieyu et al.^58^, Add a new input to the traditional Cobb–Douglas production function, called intermediate input (FI), By taking the natural logarithm of each variable on both sides of the traditional Cobb Douglas production function function, the following model Eq. (1) is obtained:

1 $$\ln TO_{{{\text{i}},{\text{t}}}} = c + \alpha \ln LI_{{{\text{i}},{\text{t}}}} + \beta \ln {\text{CI}}_{{{\text{i}},{\text{t}}}} + \gamma \ln FI_{{{\text{i}},{\text{t}}}} + \mu_{{{\text{i}},{\text{t}}}}$$

where TO_i,t_ is the total output of the “i” company in the “t” year, This article uses the inventory change of the enterprise and the total amount of main business income to measure. LI_i,t_ is the labor input of the “i” company in the “t” year. The labor input includes not only the input of labor factors, but also the efficiency of labor output and the quality of input factors. And labor compensation can better explain the changes in labor input. Therefore, this article uses paid employee salaries (listed in the company's cash flow statement) to measure. CI_i,t_ is the capital investment of the “i” company in the “t” year, mainly refers to the total capital stock of listed enterprises, Not only includes the current and fixed assets related to the company's production and operation, but also other assets that provide guarantee services for the company's production. This article uses the year-end fixed assets (listed company's balance sheet) to measure. FI_i,t_ is the input of various other factors of the “i” company in the “t” year, This article uses purchased products and services (listed in the company's cash flow statement) to measure. “α” represents the output elasticity of labor input (LI), “β” is the output elasticity of capital input (CI), “γ”is the output elasticity of the input of other factors (FI), “μ”is the random error term. The value range of “i” is 1–52, The value range of “t”is 2015, 2016, 2017, 2018. Based on the existing data of listed enterprises, Eviews 6.0 software was used to analyze the above Cobb–Douglas production function, Regression of panel data using fixed effect methods, The residuals obtained are TFP of each listed company. The specific values of TFP of each listed company are shown in Table 1.

**Table 1:** Total factor productivity of manufacturing enterprises from 2015 to 2018.

Number	2015	2016	2017	2018	Number	2015	2016	2017	2018
1	0.0628	0.0947	0.1252	0.1375	27	0.1193	0.0602	0.0104	0.0256
2	0.1754	0.1385	0.1284	0.2979	28	0.2084	0.0312	0.1520	0.0225
3	0.1262	0.1537	0.1050	0.1139	29	0.0285	0.0573	0.0229	0.0195
4	0.1388	0.0351	0.0366	0.4280	30	0.3524	0.1057	0.1028	0.1178
5	0.0256	0.0697	0.0271	0.0489	31	0.1472	0.0538	0.1285	0.1193
6	0.1282	0.0612	0.1058	0.0189	32	0.2187	0.2056	0.0142	0.0395
7	0.1156	0.0738	0.0212	0.1296	33	0.0138	0.0532	0.0353	0.1126
8	0.1210	0.1795	0.1018	0.2416	34	0.1023	0.1305	0.0823	0.0961
9	0.0129	0.0708	0.0796	0.1035	35	0.1068	0.2176	0.1295	0.0954
10	0.0715	0.0032	0.0573	0.1168	36	0.1257	0.0357	0.1521	0.0648
11	0.1458	0.1983	0.1538	0.1798	37	0.1057	0.1529	0.1304	0.0826
12	0.1309	0.0728	0.1698	0.0918	38	0.1065	0.1282	0.0572	0.2146
13	0.1158	0.0257	0.1875	0.2076	39	0.1824	0.0854	0.0827	0.1584
14	0.0697	0.0275	0.0316	0.1852	40	0.0175	0.0955	0.1254	0.2115
15	0.0578	0.0542	0.0962	0.2452	41	0.1524	0.0958	0.0236	0.0785
16	0.1007	0.1214	0.2250	0.0386	42	0.0537	0.0175	0.1082	0.1340
17	0.1862	0.0583	0.2052	0.1049	43	0.0187	0.0259	0.1154	0.1387
18	0.0268	0.1308	0.0118	0.1624	44	0.1596	0.0218	0.0257	0.1016
19	0.1072	0.0391	0.0569	0.1308	45	0.0182	0.0485	0.0083	0.0155
20	0.1071	0.0230	0.0537	0.1618	46	0.1705	0.0595	0.0452	0.0115
21	0.0039	0.1525	0.0922	0.0720	47	0.1228	0.2092	0.1482	0.0657
22	0.0652	0.1982	0.0496	0.1031	48	0.1238	0.1018	0.0172	0.0958
23	0.1058	0.0075	0.0926	0.1895	49	0.1658	0.0935	0.2849	0.1576
24	0.0282	0.1328	0.0570	0.1391	50	0.1189	0.0248	0.1663	0.2071
25	0.0528	0.1058	0.0283	0.1057	51	0.0165	0.0356	0.1203	0.0902
26	0.2649	0.1798	0.1154	0.0573	52	0.0614	0.0925	0.0258	0.1009

#### The independent variable "environmental regulations (ER)"

Environmental regulations is a necessary way for government departments to maintain the environment. Current research is mainly measured from the following perspectives. The first is the policy perspective, which can measure the change of the “three wastes” before and after the environmental policy is formulated, use this to determine the intensity of environmental regulations^59^; Secondly, from the perspective of environmental protection costs, enterprises can use the expenses incurred in the treatment of solid waste, water and air as indicators for measuring environmental regulations^60^; Third, from the perspective of pollution intensity, the proportion of pollution emissions in industrial output value can be used as an indicator for measuring environmental regulations^61^; Fourth, from the perspective of national income, the per capita income of a region or GDP per capita (GDP) can be used as an indicator to measure the environmental regulations of the region^62^. Since this article analyzes the impact of environmental regulations on green innovation and intelligent upgrading from an enterprise perspective, Therefore, this article selects the measurement method from the perspective of environmental protection costs, and considers that the enterprise's environmental protection investment is an important form of environmental regulations, At the same time, in order to exclude the influence of the scale of the enterprise, the company’ s main business income index is introduced, The company's environmental protection investment accounts for the proportion of the company's main business income as an indicator for specific measurement of environmental regulations.

#### Intermediary variable "green innovation (GI)"

At present, the measurement of green innovation is mostly based on data analysis from the perspective of the industry as a whole, often measured by the level of energy consumption reduced during the production of new products^63^, But this method is only suitable for measuring from the perspective of the industry as a whole. Some scholars use the number of green patents of each enterprise to measure the green innovation level of the enterprise^64^. Since this paper needs to measure the level of green innovation of enterprises, this paper draws on the methods of Xu et al.^64^ to measure green innovation by the number of green innovation patents. Taking the natural logarithm of the number of green innovation patents of each listed company represents the green innovation of the enterprise. Among them, the number of green patents includes green invention patents and green utility model patents.

#### Adjustment variable "environmental dynamism (ED)"

Cheng et al.^65^ proposed that the performance fluctuation of an enterprise can approximately measure the environmental dynamism, Ghosh et al.^66^ proposed to use the adjusted industry sales revenue standard deviation to measure the dynamics of the corporate environment. On the basis of previous research, Shen Huihui and others deleted some of the content of the business income that was rising steadily, and achieved a more accurate measurement of environmental dynamism. This article refers to Huihui et al.’s^67^ measurement method of environmental dynamism, using ordinary least squares. Based on the sales revenue of each listed company in the past 5 years, the standard deviation of each listed company's abnormal sales revenue in the past 5 years is estimated. The ratio of the standard deviation to the average of the company’ s sales revenue over the last five years is taken as the unadjusted environmental dynamism (UED) of the company. Then, calculate the median of the unadjusted environmental dynamism of all enterprises in each industry in each year to obtain the annual environmental dynamism (AED) of each industry. Using the method adopted by Ghosh et al.^66^, dividing the enterprise's UED by AED of each industry, the final value of the environmental dynamism (ED) is obtained.

#### Control variables "enterprise size (ES), business age (BA), asset-liability ratio (AL)"

ES is expressed by the natural logarithm of the ending value of the total assets of listed enterprises in the manufacturing industry, BA is expressed by the natural logarithm of the time from the establishment of the listed manufacturing company to the statistical year, AL is expressed as the ratio of total liabilities and total assets of a listed manufacturing company.

### Empirical model

In order to examine the relationship between green innovation, environmental dynamism and intelligent upgrading of manufacturing enterprises, this paper refers to the method proposed by Huang and Song^68^, At the same time, considering the elimination of possible heteroscedasticity and the hysteresis of environmental regulations and green innovation^69^, the measurement model is set as follows:

Without adding the independent variable and the adjustment variable, Only considering the influence of control variables (ES, BA, AL) on GI, Establish metrology model 1 as shown below:

2 $$\ln GI_{{\text{i,t}}} = \alpha_{{\text{i,t1}}} + \beta_{11} \ln ES_{{\text{i,t}}} + \beta_{12} \ln BA_{{\text{i,t}}} + \beta_{13} \ln AL_{{\text{i,t}}} + \varepsilon_{{\text{i,t1}}}$$

where α_i,t1_ is a constant term, β_11_–β_13_ regression coefficients for control variables ES, BA, and AL, respectively. “t” is the year,” i” is the ”i” company, ε _i,t1_ is the random error term.

Based on the model 1, the independent variable ER is included in the model, Establish metrology model 2 as shown below:

3 $$\ln GI_{{\text{i,t}}} = \alpha_{{\text{i,t2}}} + \beta_{21} \ln ER_{{\text{i,t} - 1}} + \beta_{22} \ln ES_{{\text{i,t}}} + \beta_{23} \ln BA_{{\text{i,t}}} { + }\beta_{24} \ln AL_{{\text{i,t}}} + \varepsilon_{{\text{i,t2}}}$$

Without adding independent variables and adjustment variables, only consider the influence of control variables ES, BA, AL on INU, Establish metrology model 3 as shown below:

4 $$\ln INU_{{\text{i,t}}} = \alpha_{{\text{i,t3}}} + \beta_{31} \ln ES_{{\text{i,t}}} + \beta_{32} \ln BA_{{{\text{i}},{\text{t}}}} { + }\beta_{33} \ln AL_{{\text{i,t}}} + \varepsilon_{{\text{i,t3}}}$$

Based on the model 3, the independent variable ER is included in the model, and the econometric model 4 is established, as follows:

5 $$\ln INU_{{\text{i,t}}} = \alpha_{{{\text{i}},{\text{t}}4}} + \beta_{41} \ln ER_{{\text{i,t} - 1}} + \beta_{42} \ln ES_{{\text{i,t}}} + \beta_{43} \ln BA_{{\text{i,t}}} { + }\beta_{44} \ln AL_{{\text{i,t}}} + \varepsilon_{{\text{i,t4}}}$$

Based on the model 3, the intermediary variable GI is included in the model, and the econometric model 5 is established, as follows:

6 $$\ln INU_{{\text{i,t}}} = \alpha_{{\text{i,t5}}} + \beta_{51} \ln GI_{{\text{i,t} - 1}} + \beta_{52} \ln ES_{{\text{i,t}}} + \beta_{53} \ln BA_{{\text{i,t}}} { + }\beta_{54} \ln AL_{{{\text{i}},t}} + \varepsilon_{{\text{i,t5}}}$$

Based on the model 4, the intermediary variable GI is included in the model, and the econometric model 6 is established, as follows:

7 $$\begin{aligned} \ln INU_{{\text{i,t}}} & = \alpha_{{\text{i,t6}}} + \beta_{61} \ln ER_{{\text{i,t} - 1}} + \beta_{62} \ln GI_{{\text{i,t}-1}} \hfill \\ & \quad + \beta_{63} \ln ES_{{\text{i,t}}} + \beta_{64} \ln BA_{{\text{i,t}}} { + }\beta_{65} \ln AL_{{\text{i,t}}} + \varepsilon_{{\text{i,t6}}} \hfill \\ \end{aligned}$$

Based on the model 3, the intermediary variable GI and manipulated variable ED are included in the model, and the econometric model 7 is established, as follows:

8 $$\begin{aligned} \ln INU_{{\text{i,t}}} & = \alpha_{{\text{i,t7}}} + \beta_{71} \ln GI_{{\text{i,t}-1}} + \beta_{72} \ln ED_{{\text{i,t}}} + \beta_{73} \ln ES_{{\text{i,t}}} \hfill \\ & \quad + \beta_{74} \ln BA_{{\text{i,t}}} { + }\beta_{75} \ln AL_{{\text{i,t}}} + \varepsilon_{{\text{i,t7}}} \hfill \\ \end{aligned}$$

Based on the model 7, in order to check the adjustment result of the environment dynamics, Product of mediator variable green innovation and moderator variable environmental dynamism is introduced as the interaction term, and the measurement model 8 is established as follows:

9 $$\begin{aligned} \ln INU_{{\text{i,t}}} & = \alpha_{{\text{i,t8}}} + \beta_{81} \ln GI_{{\text{i,t}- 1}} + \beta_{82} \ln ED_{{\text{i,t}}} + \beta_{83} \ln GI_{{\text{i,t} - 1}} \hfill \\ & \quad \times ED_{{\text{i,t}}} + \beta_{84} \ln ES_{{\text{i,t}}} + \beta_{85} \ln BA_{{\text{i,t}}} { + }\beta_{86} \ln AL_{{\text{i,t}}} + \varepsilon_{{\text{i,t8}}} \hfill \\ \end{aligned}$$

### Descriptive statistics and correlation coefficient analysis

Before conducting hypothesis testing, this article has performed descriptive statistics and correlation analysis on each relevant variable. The results are shown in Tables 2 and 3. Among them, Table 2 provides statistics and descriptions of the mean, standard deviation, minimum and maximum values of dependent variable intelligent upgrade, independent variable environmental regulations, intermediary variable green innovation, moderating variable environmental dynamism, and control variable enterprise size , business age , asset-liability ratio. Table 3 examines the correlation between the dependent variable intelligent upgrade, independent variable environmental regulations, intermediary variable green innovation, moderating variable environmental dynamism and control variable enterprise size , business age , asset-liability ratio. The correlation coefficient between the intelligent upgrade of manufacturing enterprises and environmental regulations is 0.301, which is significant at the level of 0.01; The correlation coefficient between the intelligent upgrade of manufacturing enterprises and the environmental dynamism is 0.193, which is significant at the level of 0.05. There are different degrees of correlation between the intelligent upgrading of manufacturing enterprises and various control variables.

**Table 2:** Descriptive statistics of various variables.

Variable	Code	Mean	Standard deviation	Minimum	Maximum
Intelligent upgrade	INU	0.0216	0.0395	0.0092	0.0768
Environmental regulations	ER	0.0928	0.1175	0.0087	0.2452
Green innovation	GI	0.0246	0.0423	0.0000	1.6214
Environmental dynamism	ED	0.1016	1.3942	0.0371	0.5913
Enterprise size	ES	20.4973	1.3441	10.2959	29.8275
Business age	BA	15.2656	4.2864	1.5785	63.0494
Asset-liability ratio	AL	0.5193	2.3527	− 0.2413	141.7782

**Table 3:** Correlation analysis of various variables.

Variable	INU	ER	GI	ED	ES	BA	AL
INU	1						
ER	0.301***	1					
GI	0.297***	0.235**	1				
ED	0.193**	0.167*	0.117	1			
ES	0.032	0.192**	0.127	0.037	1		
BA	0.154*	0.101	0.102	− 0.046	0.021	1	
AL	0.126	− 0.028	0.091	0.089*	0.013	− 0.034	1

***, ** and * respectively indicate that the parameter estimation is significant at the levels of 0.01, 0.05 and 0.1.

## Empirical results and discussion

### Empirical results

Before verifying the hypothesis, this article processed the statistical data as follows: Firstly, in order to avoid the estimation bias caused by extreme values, the method of Flannery and Rangan^70^ was used to perform Winsorize treatment on the persistent variables in the model at 1% level. The second is to centralize the interaction variables in the model. The third is to avoid the possibility of multiple collinearity among various variables. The variance expansion factors of each variable in the model are analyzed. The results show that the VIF values are all less than 4 and far less than 10. Therefore, there is no multicollinearity for each variable. The fourth is to prevent problems such as cross-sectional correlation, heteroscedasticity, and timing correlation that may occur due to panel data regression, With reference to Driscoll and Kraay^71^ methods, the measurement model was estimated using the Driscoll-Kraay standard deviation.

When TFP is used as an alternative indicator for the intelligent upgrade of manufacturing enterprises, the regression results of this study are shown in Table 4. First, from Model 4, we can see that the independent variable environmental regulations positively significantly affects the intelligent upgrade of the dependent variable (*β* = 0.466, p < 0.01), hypothesis H1 is proven. Second, from Model 2, we can see that the independent variable environmental regulations positively significantly affects the green innovation of intermediary variables (*β* = 0.409, p < 0.01), hypothesis H2 is proven. Again, from model 5, we can see that the green innovation of the intermediary variable positively significantly affects the intelligent upgrade of the dependent variable (*β* = 0.423, p < 0.01), hypothesis H3 is proven. At the same time, the method of Baron and Kenny^72^ was used to verify the mediation effect. From the analysis results of Model 4 and Model 6, we can see that green innovation has a positive and significant impact on intelligent upgrading (*β* = 0.342, p < 0.01), The regression coefficient of environmental regulations on intelligent upgrading has dropped from 0.466 to 0.273, and the significant level has dropped from 1 to 5%. Therefore, green innovation has an obvious intermediary role, hypothesis H4 is proven. Finally, according to the method of Aiken and West^73^ to verify the adjustment effect, According to Model 8, the coefficient of the interaction term between green innovation and environmental dynamism is 0.308, which is significant at the level of 0.01, and R2 increases by 0.004, indicating that environmental dynamism plays a positive role in regulating between green innovation and intelligent upgrade, hypothesis H5 is proven.

**Table 4:** Regression analysis results when the dependent variable is TFP.

Variable	Model 1	Model 2	Model 3	Model 4	Model 5	Model 6	Model 7	Model 8
ER		0.409*** (3.34)		0.466*** (3.71)		0.273** (2.81)		
GI					0.423*** (3.49)	0.342*** (3.05)	0.434*** (3.49)	0.416*** (3.12)
ED							0.316*** (2.95)	0.337*** (3.04)
GI × ED								0.308*** (2.74)
ES	0.151 (1.24)	0.143 (1.32)	0.177* (1.91)	0.165* (1.72)	0.173* (1.82)	0.152 (1.42)	0.151 (1.39)	0.137 (1.01)
BA	0.142 (1.37)	− 1.13* (− 1.24)	0.296** (2.31)	− 0.275** (− 2.39)	0.281** (2.49)	0.197* (1.73)	0.261** (2.51)	0.232** (2.23)
AL	0.176* (1.85)	0.153 (1.26)	0.182* (1.94)	− 0.162* (− 1.74)	0.173* (1.67)	0.153 (1.41)	− 0.158 (− 1.17)	0.139 (1.06)
Individual factor	Control	Control	Control	Control	Control	Control	Control	Control
Time factor	Control	Control	Control	Control	Control	Control	Control	Control
Constant	0.193* (1.76)	0.186* (1.71)	0.263** (1.98)	0.169* (1.67)	0.260** (2.21)	0.234** (2.03)	0.241** (2.12)	0.192* (1.73)
R^2^	0.304	0. 291	0.316	0.329	0.309	0.302	0.347	0.351
F	25.39***	21.01***	27.62***	22.91***	26.06***	21.13***	19.84***	18.78***

***, ** and * respectively indicate that the parameter estimation is significant at the levels of 0.01, 0.05 and 0.1. The "t" value is in parentheses.

When NPS is used as an alternative indicator for the intelligent upgrade of manufacturing enterprises, the regression results of this study are shown in Table 5. Model 9 is a regression model of the control variable enterprise size , business age and asset-liability ratio to the intermediary variable green innovation, Model 10 adds independent variable environmental regulations to model 9, Model 11 is a regression model of control variables enterprise size , business age and asset-liability ratio to dependent variables intelligent upgrade, Model 12 adds independent variable environmental regulations on the basis of Model 11, Model 13 adds intermediary variable green innovation on the basis of Model 11, Model 14 adds intermediary variable green innovation on the basis of Model 12, Model 15 adds green innovation of the intermediary variable and dynamic environment of the adjustment variable on the basis of the model 11, Model 16 adds the interactive items of intermediary variable green innovation and moderating variable environment dynamics on the basis of model 15. First, through Model 12, we can see that the independent variable environmental regulations positively significantly affects the intelligent upgrade of dependent variables (*β* = 0.397, p < 0.01), hypothesis H1 is proven. Secondly, Model 10 shows that the independent variable environmental regulations positively significantly affects the intermediary variable green innovation (*β* = 0.353, p < 0.01), hypothesis H2 is proven. Again, from model 13, we can see that the green innovation of intermediary variable positively significantly affects the intelligent upgrade of the dependent variable (*β* = 0.405, p < 0.01), hypothesis H3 is proven. At the same time, the method of Baron and Kenny^72^ was used to verify the mediation effect. From the analysis results of Model 12 and Model 14, we can see that green innovation has a positive and significant impact on intelligent upgrading (*β* = 0.316, p < 0.01), The regression coefficient of environmental regulations on intelligent upgrading has dropped from 0.397 to 0.284, and the significant level has dropped from 1 to 5%. Therefore, green innovation has an intermediary role, hypothesis H4 is proven. Finally, according to the method of Aiken and West^73^ to verify the adjustment effect, According to Model 16, the coefficient of the interaction term between green innovation and environmental dynamism is 0.291, which is significant at the level of 0.05. And R2 increased by 0.006, indicating that the environmental dynamism play a positive adjustment role between green innovation and the intelligent upgrade of manufacturing enterprises, hypothesis H5 is proven.

**Table 5:** Regression analysis results when the dependent variable is NPS.

Variable	Model 9	Model 10	Model 11	Model 12	Model 13	Model 14	Model 15	Model 16
ER		0.353*** (2.94)		0.397*** (3.11)		0.284** (2.27)		
GI					0.405*** (3.31)	0.316*** (2.71)	0.413*** (3.37)	0.405*** (3.11)
ED							0.302*** (2.83)	0.316*** (2.87)
GI × ED								0.291** (2.42)
ES	0.147 (1.32)	0.132 (1.13)	0.165* (1.77)	0.155 (1.42)	0.159 (1.45)	0.162* (1.67)	0.142 (1.24)	0.125 (0.98)
BA	0.172* (1.86)	− 0.220** (− 1.98)	0.285** (2.28)	− 0.167* (− 1.86)	0.271** (2.14)	0.186* (1.72)	0.249** (2.07)	0.223** (2.16)
AL	0.134 (1.53)	0.114 (1.01)	0.171* (2.16)	− 0.159 (− 1.37)	0.169* (1.85)	0.151 (1.24)	− 0.143 (− 1.04)	0.135 (1.28)
Individual factor	Control	Control	Control	Control	Control	Control	Control	Control
Time factor	Control	Control	Control	Control	Control	Control	Control	Control
Constant	0.202** (1.97)	0.198* (1.94)	0.239** (2.21)	0.228** (2.15)	0.224** (2.13)	0.234** (2.26)	0.215** (2.08)	0.207** (1.99)
R^2^	0.287	0.272	0.313	0.325	0.310	0.246	0.333	0.339
F	24.63***	21.17***	25.72***	20.15***	25.37***	22. 64***	19.11***	17.35***

***, ** and * respectively indicate that the parameter estimation is significant at the levels of 0.01, 0.05 and 0.1. The "t" value is in parentheses.

### Robustness tests

#### Endogenous test

The endogenous problems that may exist in this article are divided into two categories. One is simultaneity bias, that is, enterprises with high levels of intelligence have strong green innovation capabilities, rather than because the higher green innovation capabilities assumed in this article can promote manufacturing Enterprises are upgrading to intelligence. The second is the omission of variables. This problem often exists in empirical analysis, which may cause a simultaneous bias in the regression results of green innovation. In view of the above endogenous problems that may exist, this study simultaneously conducted the robustness test in the following two ways. On the one hand, in order to test the possible simultaneous bias problems, According to the method of Xiaogang et al.^74^, the independent variable environmental regulations and intermediary variable green innovation are lagging for two periods, and the impact of environmental regulations and green innovation on the intelligent upgrading of manufacturing enterprises is re-examined. On the other hand, in order to test the possible missing variable problem, Arellano et al.^75^ proposed that the lag value of the dependent variable already contains the missing variable. Using the lag value of the dependent variable as the control variable INUU can effectively control the influence of the missing variable. Therefore, this paper draws on the methods of Arellano et al. And uses the dynamic panel model for robustness test, and introduces the value of the dependent variable intelligent transformation and upgrade lagging one year as the control variable into the regression model.

When TFP is the dependent variable, the test results are shown in Table 6, Model 17 is a regression model of control variable enterprise size, business age and asset-liability ratio versus intermediary variable green innovation. Model 18 adds independent variable environmental regulations on the basis of model 17, Model 19 is the regression model of control variable enterprise size , business age and asset-liability ratio to dependent variable intelligent upgrade, Model 20 adds independent variable environmental regulations to model 19, Model 21 adds an intermediary variable green innovation to model 19. Model 22 adds an intermediary variable green innovation to model 20. First, from Model 20, the independent variable environmental regulations positively and significantly affects the dependent variable intelligent upgrade (*β* = 0.419, p < 0.01), hypothesis H1 is proven. Second, from Model 18, the independent variable environmental regulations positively significantly affects the intermediary variable green innovation (*β* = 0.323, p < 0.01), hypothesis H2 is proven. Again, from model 21, it can be seen that the intermediary variable green innovation positively affects the dependent variable intelligent upgrade significantly (*β* = 0.328, p < 0.01), hypothesis H3 is proven. Finally, the method of Baron and Kenny^72^ was used to verify the intermediary effect. From the analysis results of Model 20 and Model 22, we can see that green innovation has a positive and significant impact on intelligent upgrading (*β* = 0.337, p < 0.01), The regression coefficient of environmental regulations on intelligent upgrading has dropped from 0.419 to 0.271, and the significant level has dropped from 1 to 5%, therefore, green innovation has an intermediary role, hypothesis H4 is proven.

**Table 6:** Robustness test of main effect and intermediary effect when dependent variable is TFP.

Variable	Model 17	Model 18	Model 19	Model 20	Model 21	Model 22
ER		0.323*** (2.74)		0.419*** (3.42)		0.271** (2.77)
GI					0.328*** (2.91)	0.337*** (2.86)
ED						
GI × ED						
INUU	0.120 (1.16)	0.151 (1.41)	0.117 (1.05)	0.162* (1.88)	0.153 (1.39)	0.141 (1.32)
ES	0.164* (1.72)	0.116 (1.08)	0.153 (1.59)	0.137 (1.36)	0.115 (1.07)	0.123 (1.16)
BA	0.231** (2.23)	− 0.204** (− 1.98)	0.187* (1.76)	− 0.175* (− 1.69)	0.171* (1.84)	0.169* (1.71)
AL	0.168* (1.79)	0.155 (1.19)	0.164* (1.77)	− 0.167* (− 1.84)	0.152 (1.16)	0.163* (1.81)
Individual factor	Control	Control	Control	Control	Control	Control
Time factor	Control	Control	Control	Control	Control	Control
Constant	0.241** (2.03)	0.221** (2.13)	0.237** (2.07)	0.232** (2.14)	0.215** (1.99)	0.224** (2.31)
R^2^	0.284	0. 276	0.275	0. 293	0.297	0.282
F	23.16***	20.32***	25.28***	20. 41***	18.81***	19. 27***

***, ** and * respectively indicate that the parameter estimation is significant at the levels of 0.01, 0.05 and 0.1. The "t" value is in parentheses.

When NPS is the dependent variable, the test results are shown in Table 7, Model 23 is a regression model of control variable enterprise size, business age and asset-liability ratio versus intermediary variable green innovation, Model 24 adds independent variable environmental regulations based on model 23. Model 25 is the regression model of control variable enterprise size , business age and asset-liability ratio to dependent variable intelligent upgrade, Model 26 adds independent variable environmental regulations on the basis of model 25. Model 27 adds an intermediary variable green innovation on the basis of model 25. Model 28 adds an intermediary variable green innovation on the basis of model 26. First, we can see from model 26 that the independent variable environmental regulations positively affects the dependent variable intelligent upgrade significantly (*β* = 0.375, p < 0.01), hypothesis H1 is proven. Secondly, from model 24, the independent variable environmental regulations positively significantly affects the intermediary variable green innovation (*β* = 0.347, p < 0.01), hypothesis H2 is proven. Again, from model 27, it can be seen that the intermediary variable green innovation positively affects the dependent variable intelligent upgrade significantly (*β* = 0.405, p < 0.01), hypothesis H3 is proven. Finally, the method of Baron and Kenny^72^ is used to verify the intermediary effect. From the analysis results of Model 26 and Model 28, we can see that green innovation has a positive and significant impact on intelligent upgrade (*β* = 0.319, p < 0.01), The regression coefficient of environmental regulations on intelligent upgrading has dropped from 0.375 to 0.268, and the significant level has dropped from 1 to 5%. Therefore, green innovation has an intermediary role, hypothesis H4 is proven.

**Table 7:** Robustness test of main effect and intermediary effect when dependent variable is NPS.

Variable	Model 23	Model 24	Model 25	Model 26	Model 27	Model 28
ER		0.347*** (2.87)		0.375*** (3.02)		0.268** (2.13)
GI					0.405*** (3.16)	0.319*** (2.73)
ED						
GI × ED						
INUU	0.117 (1.34)	0.102 (1.15)	0.171* (1.89)	0.163* (1.73)	0.279** (2.47)	0.155 (1.42)
ES	0.125 (1.41)	0.124 (1.38)	0.175* (1.77)	0.162* (1.70)	0.119 (1.25)	0.132 (1.54)
BA	0.236** (2.38)	− 0.196* (− 1.76)	0.205** (1.97)	− 0.211** (− 2.13)	0.235** (2.18)	0.223** (2.07)
AL	0.116 (1.35)	0.107 (1.02)	0.152 (1.24)	− 0.149 (− 1.17)	0.197* (1.68)	0.187* (1.66)
Individual factor	Control	Control	Control	Control	Control	Control
Time factor	Control	Control	Control	Control	Control	Control
Constant	0.196* (1.92)	0,181* (1.83)	0.241** (2.26)	0.221** (2.12)	0.201** (2.05)	0.218** (2.09)
R^2^	0.271	0.262	0.327	0.307	0.337	0.267
F	22.07***	20.35***	26.17***	25.06***	23.81***	21. 49***

***, ** and * respectively indicate that the parameter estimation is significant at the levels of 0.01, 0.05 and 0.1. The "t" value is in parentheses.

#### Empirical results of the moderating effect

In order to test the robustness of the moderating effect, this article uses two methods to test. One is to refer to the method of Shanshi et al.^76^, remove the sample of enterprises that have been listed for less than 3 years, and use the data of the remaining listed enterprises to re-examine the moderating effect. The regression results are shown in Model 29 and Model 30 in Table 8, When TFP is the dependent variable, the regression coefficient of the interaction term between green innovation and environmental dynamism is 0.292, which is significant at the level of 0.05. It shows that the environmental dynamism have a positive moderating effect between green innovation and the intelligent upgrading of manufacturing enterprises, that is, the regression results are stable; When NPS is the dependent variable, the regression coefficient of the interaction term between green innovation and environmental dynamism is 0.277, which is significant at the level of 0.05, It shows that the environmental dynamism have a positive moderating effect between green innovation and the intelligent upgrading of manufacturing enterprises, that is, the regression results are also stable. The second is to replace the variables for testing. Referring to the method used by Luo et al.^77^, the green innovation measurement index is replaced by the R & D expenditure of the manufacturing enterprise, and the moderating effect is re-examined with the R & D expenditure data of the manufacturing enterprise. The regression results are shown in Model 31 and Model 32 in Table 8. When TFP is the dependent variable, the regression coefficient of the interaction term between green innovation and environmental dynamism is 0.239, which is significant at the level of 0.05, It also shows that the environmental dynamism have a positive moderating effect between green innovation and manufacturing enterprises' intelligent upgrade, that is, the regression results are stable; When NPS is the dependent variable, the regression coefficient of the interaction term between green innovation and environmental dynamism is 0.217, which is significant at the level of 0.05. It also shows that the environmental dynamism have a positive moderating effect between green innovation and the upgrading of manufacturing enterprises to intelligentization, that is, the regression results are also stable.

**Table 8:** Robustness test of moderating effect.

Variable	Model 29	Model 30	Model 31	Model 32
GI	0.345*** (2.63)	0.336*** (2.59)	0.355*** (2.76)	0.327*** (2.63)
ED	0.269** (2.32)	0.254** (2.28)	0.281** (2.53)	0.233** (2.01)
GI × ED	0.292** (2.04)	0.277** (2.45)	0.239** (2.11)	0.217** (1.99)
ES	0.129 (1.35)	0.115 (1.24)	0.141 (1.53)	0.105 (0.93)
BA	0.227** (2.16)	0.208** (2.07)	0.239** (2.18)	0.202** (2.05)
AL	− 0.161* (− 1.68)	0.147 (1.35)	− 0.180* (− 1.66)	0.128 (0.107)
Individual factor	Control	Control	Control	Control
Time factor	Control	Control	Control	Control
Constant	0.211** (2.32)	0.203** (2.19)	0.228** (2.29)	0.204** (2.06)
R^2^	0.275	0.261	0.287	0.266
F	15.16***	14.27***	16.35***	14.74***

***, ** and * respectively indicate that the parameter estimation is significant at the levels of 0.01, 0.05 and 0.1. The "t" value is in parentheses.

### Discussion

The relationship between environmental regulations and intelligent upgrade. The research results show that environmental regulations can actively promote the intelligent upgrade of manufacturing enterprises. This paper further refines the relevant research on environmental regulations on the transformation and upgrading of manufacturing enterprises. Intelligent upgrading is an important direction for green development of manufacturing enterprises. Existing studies have analyzed the impact of environmental regulations on the sustainable development of industry^78,79^, and the impact of environmental regulations on the sustainable development of enterprises^80^. This article introduces intelligent upgrading into the research framework. Further deepen the research on environmental regulations on the green sustainable development of manufacturing enterprises.

The relationship between environmental regulations and green innovation. The research results show that environmental regulations can actively promote green innovation in manufacturing enterprises. Li et al.^81^ divided environmental regulations into three categories: command-controlled environmental regulations, market-driven environmental regulations and voluntary environmental regulations. Taking Chinese enterprises as an example to verify, all three types of environmental regulations can positively affect enterprises' green technological innovation. Although the research results of this paper are similar to Li's conclusions, environmental regulations will not only increase the benefits of innovation for enterprises, but also increase the production costs of enterprises. The research results of different research methods and samples on environmental regulations and green innovation are not completely consistent. This result obtained in this article may be due to the relatively large size of listed enterprises in Chinese manufacturing enterprises and strong environmental awareness. Therefore, from the perspective of listed enterprises, Chinese manufacturing enterprises can make full use of environmental regulations to bring more benefits to the enterprises themselves.

The mediating effect of green innovation. The research results show that green innovation can actively promote the intelligent upgrade of manufacturing enterprises. At the same time, green innovation can play an intermediary role between environmental regulations and the intelligent upgrading of manufacturing enterprises. In other words, while other factors remain unchanged, green innovation can promote the intelligent upgrade of manufacturing enterprises. The stronger the green innovation capability, the more beneficial to the intelligent upgrading of manufacturing enterprises. At the same time, environmental regulations indirectly affect the intelligent upgrade of manufacturing enterprises through green innovation. The research results of this paper are not exactly the same as the path of environmental regulations proposed by Zhai^82^ on the green transformation of manufacturing industry. We explored the impact of green innovation on the intelligent upgrading of manufacturing enterprises, and revealed the intermediary role of green innovation between environmental regulations and the intelligent upgrading of manufacturing enterprises.

The moderating effect of environmental dynamism. Environmental dynamism have a positive adjustment effect between green innovation and the intelligent upgrading of manufacturing enterprises. With green innovation remaining unchanged, the higher the environmental dynamism, the greater the positive impact of green innovation on the intelligent upgrade of manufacturing enterprises. Chan^44^ believes that environmental dynamism positively regulate the relationship between green product innovation and corporate performance. The conclusion of this article not only deepens related research. Moreover, it analyzes the interrelationship between environmental dynamism, green innovation and intelligent upgrading of manufacturing enterprises. Through empirical analysis, it is concluded that environmental dynamism can positively regulate the relationship between green innovation and the intelligent upgrade of manufacturing enterprises.

## Conclusion and implications

### Conclusion

Based on the relevant theories of "Industry 4.0", this paper analyzes the relationship between environmental regulations, green innovation, environmental dynamism and the intelligent upgrading of manufacturing enterprises. The hypothesis of the relationship between environmental regulations, green innovation, intelligent upgrading of manufacturing enterprises and environmental dynamism is proposed. Taking the listed enterprises in the intelligent manufacturing pilot enterprises determined by the Ministry of Industry and Information Technology of China as the research object, the relevant statistical data of manufacturing enterprises from 2015 to 2018 were collected for empirical analysis. The conclusions are as follows: In the process of intelligent upgrading of manufacturing enterprises, environmental regulations are positively affecting the intelligent upgrading of manufacturing enterprises. Environmental regulations are positively affecting green innovation in manufacturing enterprises. Green innovation is positively affecting the intelligent upgrading of manufacturing enterprises. Green innovation has an intermediary role between environmental regulations and the intelligent upgrading of manufacturing enterprises. Environmental dynamism positively regulate the relationship between green innovation and the intelligent upgrading of manufacturing enterprises. Specifically, when the environmental dynamism are high, the positive impact of green innovation on the intelligent upgrading of manufacturing enterprises is relatively strong, when the environmental dynamism are low, the positive impact of green innovation on the intelligent upgrading of manufacturing enterprises is relatively weak.

### Management implications

Taking environmental regulations as an entry point, the article analyzes and examines the relationship between environmental regulations, green innovation, intelligent upgrading of manufacturing enterprises and environmental dynamism. At the same time, it has enriched and expanded relevant research on the intelligent upgrading of manufacturing enterprises. In addition, from the perspective of environmental dynamism, it analyzes the importance of changes in internal and external environments and market demands for the intelligent upgrade of manufacturing enterprises. The research conclusion can provide the following theoretical revelation for the transformation and upgrading of manufacturing enterprises to intelligentization.

First of all, In the past, many factors influencing the intelligent upgrade of manufacturing enterprises were studied from the perspectives of digital integration, interconnection and intelligent technology. However, environmental regulations and green innovation also occupy an important position in the process of manufacturing enterprises upgrading to intelligence. Based on the relevant content of the "Industry 4.0" theory, this article clarifies the promotion of environmental regulations and green innovation on the intelligent upgrading of manufacturing enterprises. It is conducive to deepening the understanding of the relationship between environmental regulations and intelligent upgrading of manufacturing enterprises, green innovation and intelligent upgrading of manufacturing enterprises. It is also promote further research on related theories.

Secondly, the intermediary role of green innovation shows that the promotion of environmental regulations on intelligent upgrading can also be achieved through green innovation. Green innovation can not only directly affect intelligent upgrades, but also act as an intermediary variable to promote the intelligent upgrade of manufacturing enterprises together with environmental regulations. Manufacturing companies should pay full attention to the important relationship between green innovation and intelligent upgrading, and promote the intelligent upgrading of enterprises through efforts to achieve green innovation. In view of this, manufacturing enterprises can start from the perspective of green innovation capability when promoting intelligent upgrading, and promote the intelligent upgrading of manufacturing enterprises by enhancing the green innovation capability of manufacturing enterprises.

Finally, due to the limited resources of the manufacturing enterprise, how to rationally allocate various resources within the manufacturing enterprise so as to improve the quality and efficiency of the manufacturing enterprise's intelligent upgrade. It has always been a difficult point for manufacturing enterprises to implement intelligent transformation and upgrading. Introducing environmental dynamism, this paper proposes that environmental dynamism can positively regulate the relationship between green innovation and the intelligent upgrading of manufacturing companies. It reveals the unique role of environmental dynamism in the intelligent upgrading of manufacturing enterprises. The article links the uncertainty of the dynamic environment with the intelligent upgrading of manufacturing enterprises. It provides a reference direction for finding more factors that affect the intelligent upgrade of manufacturing enterprises in the future. When manufacturing enterprises are upgrading intelligently, they should make an objective and accurate assessment of the dynamic environment such as the internal and external environment and market demands they are currently facing. In order to grasp and make good use of the intelligent development opportunities brought by the highly dynamic environment, to improve the enterprise's intelligence level faster and better, and to complete the enterprise's established intelligent upgrade goals.

### Limitation and further research

This article starts from "Industry 4.0" and green development theory, we only studied the relationship between environmental regulations, green innovation, environmental dynamism and the intelligent upgrading of manufacturing companies. However, the impact of other factors on the intelligent upgrade of manufacturing companies has not been fully considered. In the future, consider including other factors into the unified analytical framework. Establish the theory model of intelligent upgrading of manufacturing enterprises to comprehensively analyze the influence mechanism of various factors on the intelligent upgrading of manufacturing enterprises.
